# Highly Efficient Synthesis of Chlorogenic Acid Oleyl Alcohol Ester under Non-Catalytic and Solvent-Free Conditions

**DOI:** 10.3390/molecules28093948

**Published:** 2023-05-08

**Authors:** Cong Sun, Hui Liu, Yanran Chen, Xianzhi Wei, Shaohua Liang

**Affiliations:** 1 Grain, Oil and Food Engineering Technology Research Center of the State Grain and Reserves Administration, Key Laboratory of Henan Province, Henan University of Technology, Lianhua Road 100, Zhengzhou 450001, China; suncong0511@haut.edu.cn; 2 College of Food Science and Engineering, Henan University of Technology, Lianhua Road 100, Zhengzhou 450001, China; 18838910903@163.com (H.L.); mccyr@163.com (Y.C.); wyweixianzhi@163.com (X.W.)

**Keywords:** chlorogenic acid, chlorogenic acid oleyl alcohol ester, esterification, non-catalytic synthesis

## Abstract

As a natural polyphenolic compound, chlorogenic acid (CGA) has attracted increasing attention for its various biological activities, such as antioxidant, liver protection, intestinal barrier protection, and effective treatment of obesity and type II diabetes. However, the poor solubility of CGA in hydrophobic media limits its application in the food, drug and cosmetic industries. In order to obtain new hydrophobic derivatives, a highly efficient synthesis approach of CGA oleyl alcohol ester (CGOA) under non-catalytic and solvent-free conditions was developed in this study. The influences of reaction temperature, reaction time, substrate molar ratio, and stirring rate on the CGA conversion were investigated. The results showed that the optimal conditions were as follows: reaction temperature 200 °C, reaction time 3 h, molar ratio of CGA to oleyl alcohol 1:20, and stirring rate 200 rpm. Under these conditions, the CGA conversion could reach 93.59%. Then, the obtained crude product was purified by solvent extraction and column chromatography, and the purify of CGOA was improved to 98.72%. Finally, the structure of CGOA was identified by FT-IR, HPLC-MS and NMR. This study provides a simple and efficient strategy for the preparation of CGOA with the avoidance of catalysts and solvents.

## 1. Introduction

Synthetic phenolic antioxidants, such as butylated hydroxytoluene and butyl hydroxyanisole, have been used to delay the process of lipid oxidation. However, due to its potential carcinogenicity, more and more attention has been paid to natural antioxidants [1]. Polyphenol compounds, which are naturally present in various foods, shrubs and medicinal plants (such as tea, coffee, vegetables, cereals and so on), have been widely used for antioxidant, anti-inflammation, antibiosis, and cancer treatments [2]. Chlorogenic acid (CGA) is a kind of polyphenolic compound with antioxidant properties, which is rich in foods and herbs such as peaches, sweet potato, coffee beans, apples, grapes, eucommia, tea and so on [3,4,5,6]. In addition, CGA has been proven to have a variety of health functions, such as antioxidant, liver protection, intestinal barrier protection, and effective treatment of obesity and type II diabetes [7,8,9,10,11]. According to Zang et al. [12], CGA could strongly scavenge ·OH and protect endogenous antioxidants from being depleted.

Although CGA exhibits many biological activities, its application is greatly restricted in the food, drug and cosmetic industries because of its poor solubility in hydrophobic media. To date, esterification modification by aliphatic molecules has been used as a major tool to alter solubility in oil-based formulae and emulsions, which mainly includes enzymatic method and chemical method [13,14]. In recent years, there have been many studies on the enzymatic synthesis of hydrophobic derivatives of phenolic acid [15,16]. Zhu et al. reported that Lipozyme RM exhibited a highly efficient regioselective acylation of CGA, and was expected to be an alternative enzyme for Novozym 435 due to its cheaper price and better selectivity for CGA modification. Under the optimal conditions with the molar ratio of CGA to acyl donor of 1:10 at 55 °C under shaking (400 rpm), the conversion rates of CGA were 59.6% (after 7 days of reaction) and 46.0% (after 2 days of reaction) using vinyl butyrate and vinyl octanoate as the acyl donors, respectively [15]. It is generally characterized by low solvent consumption, environmentally friendly processes, and high purity products, whereas high cost and low catalytic efficiency limits its wide applications. Compared with enzymatic catalysis, chemical catalysis is more effective, lower costing, and has a wide range of sources. Xiang and Ning reported that the reaction of CGA with lauric acid in a non-aqueous phase catalyzed by triethylamine (TEA) was carried out at 35 °C for only 4 h, which greatly shortened the reaction time compared with enzymatic method [17]. In our previous study [14], chlorogenic oleate was synthesized by the acyl chloride method using TEA as the catalyst and N,N-dimethylmamide (DMF) as the solvent, and a CGA conversion of 76.62% was achieved with the molar ratio of CGA to oleoyl chloride of 1:1.5 and the molar ratio of CGA to TEA of 1:1.5 at a temperature of 25 °C with a reaction time of 20 min by adding method of oleoyl chloride via 4 aliquots. However, the reaction process is unselective, resulting in many side reactions and difficult separation [18]. These limitations of catalytic esterification promote the exploration and development of non-catalytic esterification.

In the non-catalytic process, the separation step of catalysts can be omitted, and therefore, the product is not polluted, which can overcome the shortcomings of the existing homogeneous or heterogeneous catalyst processes and ensure the product quality [19,20]. A non-catalytic process has been applied in many esterification reactions, such as the synthesis of phytosterol esters, glycerides, and biodiesel [19,21,22,23]. As far as we know, it has not been used in the esterification of CGA, and most of the studies on the esterification of CGA were carried out by enzymatic method with a relatively long reaction time and low conversion. Giraldo et al. reported that various fatty chlorogenate esters were obtained after 96 h with 61–93% yields [24]. In another study, the acylation reaction of CGA (extracted from *Hydrangea macrophylla*) with palmitic acid under the catalysis of Novozym 435 was carried out, and the main product 4-O-palmitoyl chlorogenic acid and the minor product 3-O-palmitoyl chlorogenic acid were obtained with the conversion rate between 14–60% in 7 days [25]. In theory, with the increase in the chain length of acyl donor, the lower the esterification rate of CGA, the higher the lipophilicity of the esterified product [24,26]. Therefore, the development of an efficient method for CGA ester production is urgently required.

The present study aimed to establish a highly efficient approach for synthesizing CGA oleyl alcohol ester (CGOA) in a non-catalytic and solvent-free system. The effects of reaction temperature, reaction time, molar ratio of CGA to oleyl alcohol, and stirring rate on the conversion of CGA were investigated. The target product was then isolated by solvent extraction and column chromatography. Finally, the structure of CGOA was characterized by high performance liquid chromatography-mass spectrometer (HPLC-MS), fourier transform infrared (FT-IR) spectroscopy, and nuclear magnetic resonance (NMR).

## 2. Results and Discussion

### 2.1. Influence of Reaction Parameters

#### 2.1.1. Influence of Reaction Temperature

**Figure 1:**
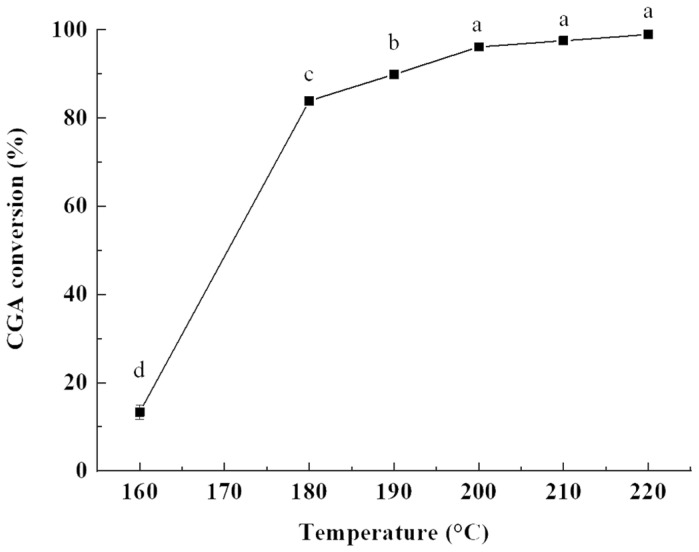
Effect of reaction temperature on the CGA conversion. Reaction conditions: molar ratio of CGA to oleyl alcohol, 1:20; stirring rate, 600 rpm; and reaction time, 4 h. Results are presented as means ± standard deviations of triplicate measurements. Different letters in the data plot indicate significant differences (*p* < 0.05).

As shown in Figure 1, the conversion of CGA sharply increased from 6.66% to 83.91% when the reaction temperature increased from 160 °C to 180 °C, which indicated that the increase in reaction temperature was beneficial to promote the esterification reaction. Due to the large steric hindrance of the reaction between CGA and oleyl alcohol and in a catalyst-free system, the reaction should be carried out at high temperature [13,24,26]. In theory, esterification reaction needs to break the bond before esterification, which needs to endothermic. Due to the large molecular structure of CGA, the bond is difficult to break below 160 °C, which is not conducive to the esterification reaction. As the temperature increases, it reaches the energy required for bond breaking, which is beneficial to esterification and the CGA conversion. In addition, this reaction is a solid–liquid reaction system, and increasing temperature will reduce the viscosity of the reaction mixture and promote the reaction [27]. As the temperature continued to increase to 200 °C, the CGA conversion reached 96.12%, and the esterification reaction reached equilibrium. Considering that an excessive temperature is easy to cause unnecessary side reactions (such as polymerization) and energy waste, 200 °C was selected as the optimal reaction temperature [28].

#### 2.1.2. Influence of Reaction Time

**Figure 2:**
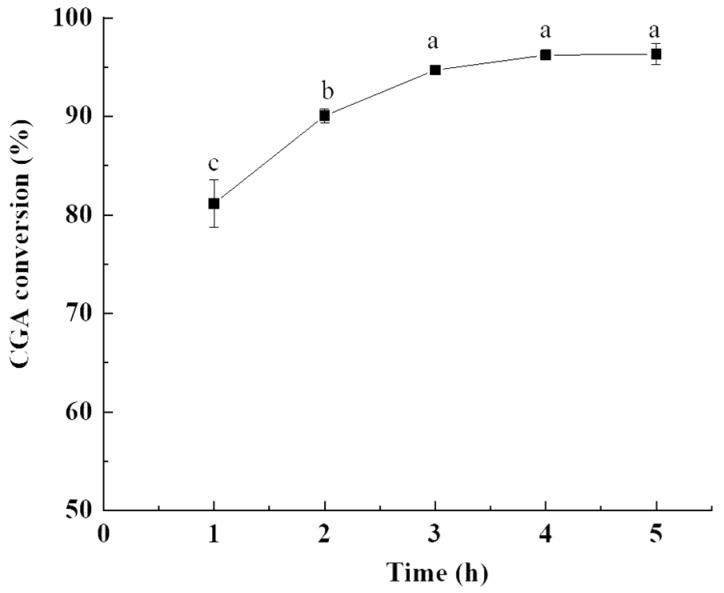
Effect of reaction time on the CGA conversion. Reaction conditions: molar ratio of CGA to oleyl alcohol, 1:20; stirring rate, 600 rpm; and reaction temperature, 200 °C. Results are presented as means ± standard deviations of triplicate measurements. Different letters in the data plot indicate significant differences (*p* < 0.05).

The influence of reaction time on the CGA conversion was shown in Figure 2. The CGA conversion increased with reaction time during 1–3 h and reached equilibrium during 3–5 h (96.32%). A similar trend was consistent with that of Panchal et al. [29]. At the initial stage of the reaction, sufficient substrates and the high probability of intermolecular collision are favorable for esterification, which leads to the rapid increase in CGA conversion rate. With the extension of reaction time, the substrates are consumed and the reaction reached becomes balanced. When the reaction time exceeds the optimal time, the conversion of CGA may be reduced due to the backward transesterification reaction [30]. Varma et al. [31] found that the kinetic equilibrium was achieved in 2–3 h with the conversion of 37% and 53%, respectively for isoamyl laurate and isoamyl stearate. Guyot et al. [32] and Stamatis et al. [33] reported the enzymatic esterification of phenolic acids and fatty alcohols, and a long reaction time (at least 1 d) in both cases was needed to obtain reasonable yields. Twu et al. [34] esterified hydroxyphenylpropionic acid and octanol with 95.9% molar conversion in 58.2 h. Overall, the optimal reaction time for CGOA preparation was 3 h.

#### 2.1.3. Influence of Molar Ratio of CGA to Oleyl Alcohol

**Figure 3:**
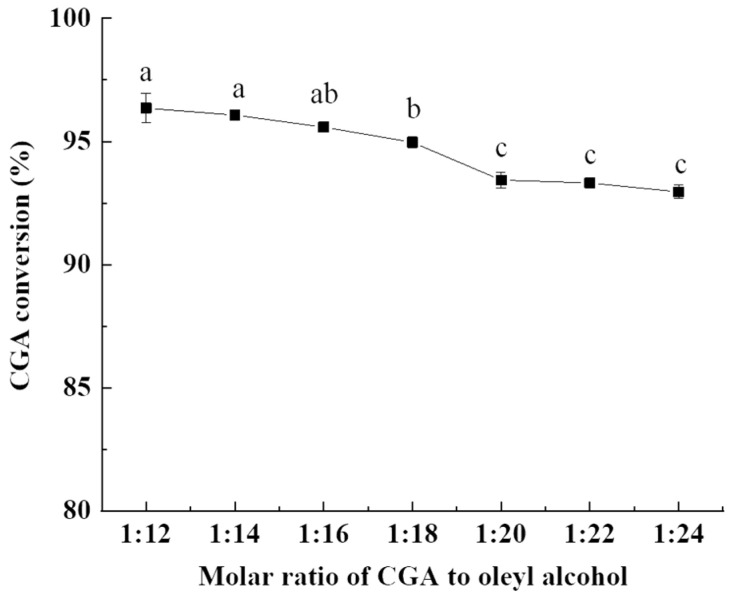
Effect of molar ratio of CGA to oleyl alcohol on the CGA conversion. Reaction conditions: reaction time, 4 h; stirring rate, 600 rpm; and reaction temperature, 200 °C. Results are presented as means ± standard deviations of triplicate measurements. Different letters in the data plot indicate significant differences (*p* < 0.05).

One challenge in a solvent-free system, which is not as problematic in solvent-added systems, is to ensure that the reagents are effectively mixed [35]. This experiment is a solid–liquid reaction system, and the density of CGA solids is small. If the amount of oleyl alcohol added is too low, the solid content in the system is the main part, which is not beneficial to the dispersion of the system and the contact collision between molecules, and may cause local overheating. Therefore, based on the results of our pre-experiments, the influence of molar ratio of CGA to oleyl alcohol from 1:12 to 1:24 on the CGA conversion was evaluated in Figure 3.

As the ratio of oleyl alcohol increased, the CGA conversion slowly decreased to a stable trend as shown in Figure 3. There were no significant differences in the CGA conversion when the substrate molar ratio was 1:12–1:16, which was the same as 1:20–1:24. These results agreed with the previous studies [29,36]. Although the highest conversion was observed when the substrate molar ratio was 1:12, the CGA solid was not well dispersed in oleyl alcohol during the reaction process, and there was a considerable amount of the CGA solid deposited at the bottom. This phenomenon might lead to local overheating and promoting the self-esterification reaction of CGA, resulting in an increased conversion of CGA [37]. When the substrate molar ratio was 1:20, it could be observed that the CGA solid was more uniformly dispersed in oleyl alcohol, which was beneficial to the esterification reaction and could inhibit the self-esterification reaction of CGA. Guyot et al. [32] suggested that excessive ethanol could effectively stir the reaction medium and appropriately disperse the insoluble substances in the case of esterification reaction without the solvent added. Chen et al. [38] synthesized hexyl dihydrocaffeate using p-toluenesulfonic acid as a catalyst under the following conditions: molar ratio of 1:30 (dihydrocaffeic acid to hexanol) and 80 °C for 2 h with the yield of 99.3%. Stamatis et al. [33] increased the molar ratio of ascorbic acid to myristic acid from 1:1 to 1:15, and the conversion rate increased from 30% to 65%. However, when the ratio of oleyl alcohol is too high, the probability of intermolecular collision is reduced, which is not conducive to the esterification reaction and subsequent separation and purification. Therefore, the molar ratio of CGA to oleyl alcohol was 1:20, which was considered to be suitable and economically feasible for the CGOA production.

#### 2.1.4. Influence of Stirring Rate

**Figure 4:**
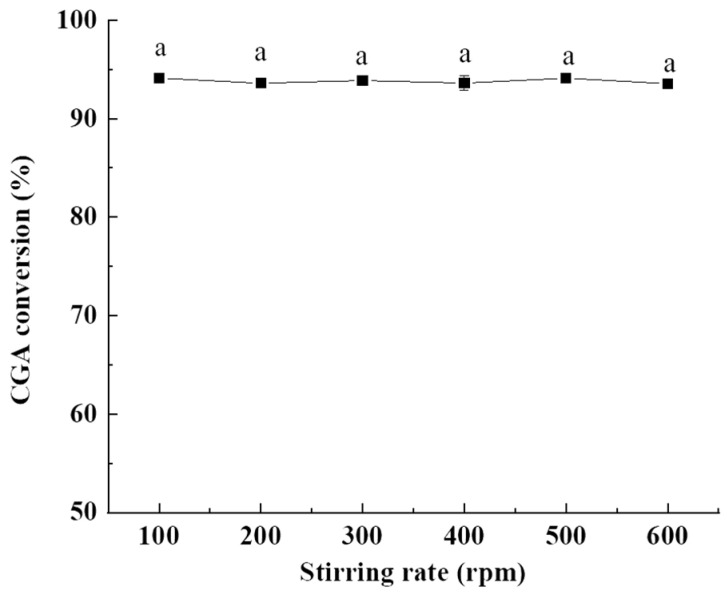
Effect of stirring rate on the CGA conversion. Reaction conditions: molar ratio of CGA to oleyl alcohol; 1:20, reaction temperature, 200 °C; and reaction time, 4 h. Results are presented as means ± standard deviations of triplicate measurements. Different letters in the data plot indicate significant differences (*p* < 0.05).

This reaction system is a solid–liquid heterogeneous system, and previous studies have reported that vigorous mixing was quite necessary to increase the contact area between two immiscible phases [39,40]. However, there was no significant difference in the CGA conversion with the increase in stirring rate, and the CGA conversion was approximately maintained at about 93–94% (Figure 4). These results might be caused by the fact that the viscosity of oleyl alcohol was greatly reduced under high temperature and high vacuum conditions, which made CGA and oleyl alcohol easy to evenly mix. Therefore, high reaction rate could be achieved under a slow stirring rate. In addition, 200 °C has almost reached the boiling point of oleyl alcohol, and the esterification reaction also produces water. Thus, slight boiling was observed in the experiment, which could also promote the contact and reaction between molecules. Considering the mixing uniformity and energy saving, 200 rpm was selected as the optimal stirring rate, which was in accordance with Panchal et al. [39].

### 2.2. Purification of CGOA

Due to the excessive oleyl alcohol in the reaction mixture, the crude CGOA product was first extracted via solvent extraction. According to the method described by Giraldo et al. [24], the mixture was extracted with n-hexane/acetonitrile/water, and it was found that the emulsification was difficult to further separate. Thus, this method was not suitable for this study, which might be due to the differences in the acyl donors or reaction conditions. After comparing different extractants, it was found that the problem of intermediate emulsification could be solved by replacing *n*-hexane with petroleum ether. Then, the ratio of different extractants was further adjusted and the crude product, an oily brown solid, was obtained. Finally, the crude product was further purified by column chromatography and eluted with toluene/isopropanol (6/1, *V*/*V*) to obtain a brown paste product. The obtained CGOA was dissolved in methanol, and its purity was 98.72% by HPLC, which indicated that the unreacted oleyl alcohol was removed effectively. The highly purified CGOA was utilized in the following structural characterization.

### 2.3. Identification of CGOA

#### 2.3.1. HPLC-MS Analysis of CGOA

**Figure 5:**
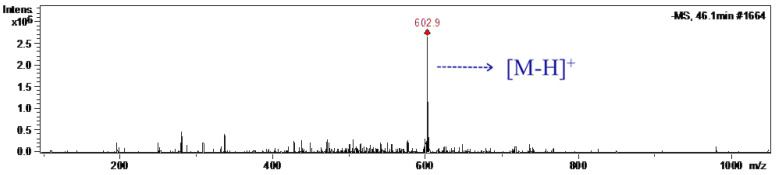
MS spectra of the purified CGOA.

The mass spectra of the purified CGOA were obtained in a MS spectrometer with negative ESI mode (Figure 5). The characteristic fragment ion of [M-H]^−^ at *m*/*z* 602.9 was observed. Theoretically, the relative molecular weight for CGOA was 604, which indicated that the sample existed as CGOA.

#### 2.3.2. FT-IR Analysis of CGOA

**Figure 6:**
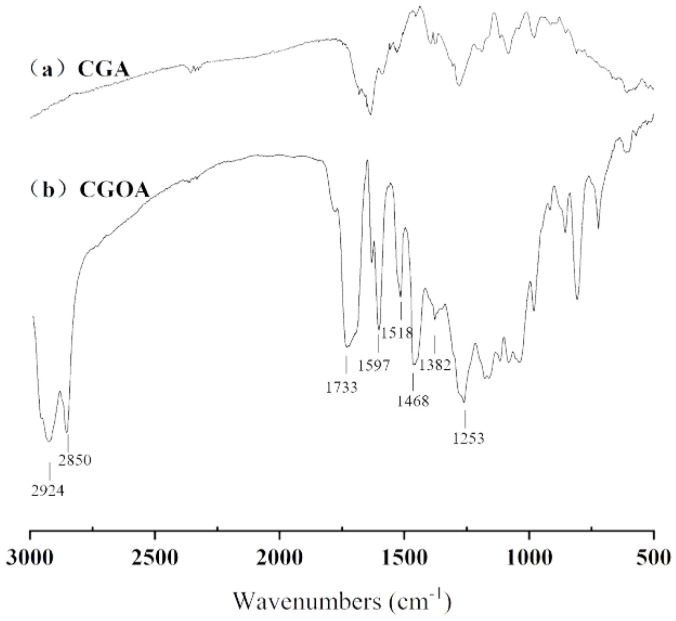
FT-IR spectra of CGA (**a**) and CGOA (**b**).

The molecular structures of CGA and CGOA were identified by FT-IR (Figure 6). The peaks at 2924 and 2850 cm^−1^ were the characteristic peaks of -CH_3_ and six-membered ring, respectively. The strong absorption peak at 1733 cm^−1^ represented the stretching vibration of C=O. The peaks at 1597, 1518, and 1468 cm^−1^ suggested the presence of C=C. The peak at 1382 cm^−1^ resulted from the deformation vibration of OH in-plane. The band at 1253 cm^−1^ suggested the presence of long-chain fatty alcohol esters. The bands in a range of 720–660 cm^−1^ resulted from the bending vibration signals of C-H in the long-chain methylene group. Compared with the CGA, the stretching vibration peak of CGOA at 2950–2850 cm^−1^ was obviously strengthened, and the band at 1733 cm^−1^ was found, which implies the existence of the carbonyl group of a new ester group [17,26,41]. These results revealed that oleyl alcohol group was successfully inserted into CGA, and CGOA were successfully synthesized.

#### 2.3.3. NMR Analysis of CGOA

The NMR spectra and chemical structure of CGOA were shown in Figures S1–S3 (Supplementary Materials). The profile of CGOA was shown as follows: ^1^H NMR (500 MHz, MeOD) δ 7.54 (s, 1H, -CH=C**H**-), 7.07 (d, *J* = 2.0 Hz, 1H, -C=C**H**-), 6.96 (d, *J* = 2.0 Hz, 1H, =CH-C**H**=), 6.80 (s, 1H, -C=C**H**-), 6.22 (s, 1H, -C**H**=CH-), 5.36 (d, *J* = 0.9 Hz, 3H, -O-C**H**-, -C**H**=C**H**-), 4.12 (m, *J* = 3.5 Hz, 1H, HO-CH-), 3.76 (q, *J* = 3.4 Hz, 1H, HO-CH-), 3.56 (s, 2H, -C**H**_2_-CH_2_-), 2.21 (d, *J* = 5.6 Hz, 2H, -C**H**_2_-), 2.04 (s, 6H, 3 × -C**H**_2_-), 1.56 (s, 2H, -C**H**_2_-), 1.33 (s, 22H, 11 × -C**H**_2_-), 0.92 (s, 3H, -C**H**_3_). ^13^C NMR (150 MHz, MeOD) δ 173.63 (C), 166.80 (C), 148.34 (C), 145.86 (C), 145.50 (CH), 129.45 (CH), 126.20 (C), 121.58 (CH), 115.12 (CH), 113.74 (CH), 99.99 (C), 74.24 (CH), 70.82 (CH), 65.29 (CH), 61.61 (CH_2_), 36.62 (CH_2_), 32.28 (CH_2_), 31.67 (CH_2_), 26.72 (CH_2_), 25.54 (CH_2_), 22.35 (CH_2_), 13.07 (CH_3_). 

Compared with the chemical shifts of CGA, there was little change among the 5 groups of protons (1′–9′) on the benzene ring and conjugated double bonds within the range of 6.2–7.6 ppm, indicating that CGOA maintains the 1′–9′ skeleton structure of CGA (Figure S1, Table S1, Supplementary Materials). In addition, the classic six groups of proton signal peaks of oleyl alcohol appeared in the 1H NMR of CGOA. According to Figure S2 and Table S1 (Supplementary Materials), the results of the 13C spectrum were consistent with those of the 1H spectrum.

## 3. Materials and Methods

### 3.1. Materials

CGA standard (purity > 95%) was supplied by National Institute for the Control of Pharmaceutical and Biological Products (Beijing, China). CGA (purity > 95%) was supplied by Hunan Jiamu Biological Technology Co., Ltd. (Changsha, China). Oleyl alcohol (purity of 82.86%) and iodine were purchased from Aladdin Reagent Co., Ltd. (Shanghai, China). Deuterated methanol was purchased from Macleans Reagent Company (Shanghai, China). Methanol was HPLC grade and purchased from Changde Chengwei Trading Co., Ltd. (Changde, China). All other chemicals and solvents were analytical grade and purchased from Tianjin Kemiou Chemical Reagent Co., Ltd. (Tianjin, China).

### 3.2. Preparation of CGOA

A certain amount of CGA and oleyl alcohol were stirred and dehydrated in a round bottom flask at 90 °C and in a vacuum above 0.095 MPa for 30 min. Then, the esterification was carried out under the condition of the vacuum above 0.098 MPa.

The reaction conditions of substrate molar ratio (CGA/Oleyl alcohol, 1:12, 1:14, 1:16, 1:18, 1:20, 1:22, 1:24), reaction temperature (160, 180, 190, 200, 210 and 220 °C), reaction time (1, 2, 3, 4 and 5 h), and stirring rate (100, 200, 300, 400, 500 and 600 rpm) were varied for optimization. The CGA conversion was used as an evaluation index.

### 3.3. HPLC Analysis

The reaction mixture was dissolved with methanol. A certain amount of the solution was accurately weighed into a volumetric flask, and the volume was fixed with methanol. Then, the solution was filtered with a 0.22 μm polypropylene filter for HPLC analysis.

HPLC analysis was carried out according to the procedures of Hernandez et al. with minor modifications [13]. CGA was measured by Waters E2695 HPLC using an Agilent ZORBAX 300SB-C18 column (5 µm, 4.6 × 250 mm, Agilent, Santa Clara, CA, USA) at a flow rate of 0.8 mL∙min^−1^. Detection wavelength was set at 327 nm. Column temperature was set at 30 °C. The separation of CGA was carried out using a binary solvent gradient program of methanol (A) and 1.0% acetic acid solution (B). The proportion of elution A increased from 5% to 75% in 35 min, then increased to 100% in 40 min, and returned to 5% in 50 min and was maintained for 10 min.

The concentration of CGA was measured using the external standard method. CGA conversion rate was calculated as follows:

$$\mathrm{CGA}\;\mathrm{conversion}\;(\%)=\frac{m_{1}\times 0.9588-m_{2}}{m_{1}\times 0.9588}\times 100\%$$

$$m_{2}=\frac{c\times 5\times m_{3}}{m_{4}\times 1000}$$

where *m*_1_ and *m*_2_ are the CGA mass before reaction and after the reaction, respectively (g); 0.9588 is the purity of CGA; *c* is the concentration of CGA (μg·mL^−1^) and 5 is the sample volume (mL); *m*_3_ is the mass of the solution dissolved in methanol after the reaction (g); and *m*_4_ is the mass of methanol solution after the reaction (g), which is weighed quantitatively.

### 3.4. Isolation and Purification of CGOA

This method was performed as described by Giraldo et al. with some modifications [24]. The reaction product was firstly extracted by the mixed solvents (petroleum ether/acetonitrile/water = 4/3/1, *V*/*V*/*V*), and the petroleum ether phase was extracted twice by acetonitrile/water (3/1, *V*/*V*). All the acetonitrile/water phases were combined and evaporated to obtain the crude product. Then, the sample was separated by column chromatography and eluted with toluene/isopropanol (6/1, *V*/*V*). The fractions were collected in test tubes. The solvents were removed by rotary evaporation and vacuum drying to obtain the pure CGOA.

### 3.5. HPLC-MS Analysis

The purified CGOA was characterized by HPLC-MS (Agilent 1200). The negative-ion electrospray ionization mode was used at optimized conditions, as follows: mass range, 100–1000 *m*/*z*; drying temperature, 350 °C; nebulizer pressure, 40 psi; and drying gas flow, 8.0 L·min^−1^. The HPLC conditions were the same as 3.3.

### 3.6. FT-IR Analysis

CGA and the purified CGOA were pressed with KBr and scanned by FT-IR (WQF-510). The spectra were recorded in 16 scans, and the resolution was 4 cm^−1^ between 3000 cm^−1^ and 500 cm^−1^.

### 3.7. NMR Analysis

^1^H NMR and ^13^C NMR analyses were performed using the purified CGOA dissolved in deuterated methanol. All NMR measurements were performed on a Bruker NMR spectrometer (Avance III HD 500M, Faellanden, Switzerland). The ^1^H and ^13^C NMR spectra were acquired at 500 and 150 MHz with a 5 mm probe and temperature of 298 K, respectively. Spectrum widths of ^1^H NMR and ^13^C NMR were 10,000 and 31,250 Hz. The scans were 256 and 20,000 times, respectively.

### 3.8. Statistical Analysis

A single factor experiment was used to optimize the various reaction parameters, and the results were expressed as means ± standard deviations. All analyses of data were performed using SPSS Statistics 20 software. The differences were compared by one-way analysis of variance (ANOVA, LSD’s test), and values marked with different letters were significantly different (*p* < 0.05). 

## 4. Conclusions

Efficient, non-catalytic, and solvent-free synthesis of lipophilic derivatives is a big challenge for natural phenolic antioxidants. This study attempted to conduct the esterification reaction of CGA and oleyl alcohol at high temperature and high pressure without the use of a catalyst and solvent. The several process factors involved in the esterification reaction were investigated for impact on the conversion of CGA, and found that only reaction temperature and reaction time were significant for the esterification reaction. The optimal synthesis conditions were determined by use of single factor optimization as follows: reaction temperature, 200 °C; reaction time, 3 h; molar ratio of CGA to oleyl alcohol, 1:20; and stirring rate, 200 rpm. Under these conditions, the CGA conversion was up to 93.59%. After solvent extraction and column chromatography, CGOA purity could be further improved to 98.72%. Finally, the structure of the purified product was identified as CGOA using a combination of FT-IR, HPLC-MS and NMR.

Compared with the method in this study, although the acyl chloride method reported by our previous study requires lower reaction temperature and shorter reaction time, its CGA conversion is only 76.62% under the optimal conditions, and it requires the addition of some substances with certain toxicities (sulfoxide chloride, TEA and DMF) [14]. In addition, compared with the enzymatic methods [15,25], this non-catalytic, and solvent-free method greatly improved the CGA conversion and reduced the reaction time and production cost. Therefore, a highly efficient synthesis approach of CGOA under non-catalytic and solvent-free conditions has been successfully developed in this study. The preparation of CGOA will provide a new opportunity for CGA and its esters in food, cosmetic, and medicine application.

## Data Availability

Not applicable.

## Supplementary Materials

The following supporting information can be downloaded at: https://www.mdpi.com/article/10.3390/molecules28093948/s1, Figure S1: The 1H NMR spectra of CGOA; Figure S2: The ^13^C NMR spectra of CGOA; Figure S3: The chemical structure of CGOA; Table S1: NMR characteristics of the 1H and 13C of CGA and CGOA.

## Sample Availability

Samples of the compounds are available from Shaohua Liang on valid request.
