# Supplementary material for: Highly Efficient Synthesis of Chlorogenic Acid Oleyl Alcohol Ester under Non-Catalytic and Solvent-Free Conditions
Source: Molecules. 2023 May 8;28(9):3948. doi: 10.3390/molecules28093948 (PMC10180084; doi:10.3390/molecules28093948)
Supplement: Supplementary file 1 [file molecules-28-03948-s001.zip › molecules-2364345-supplementary.pdf]

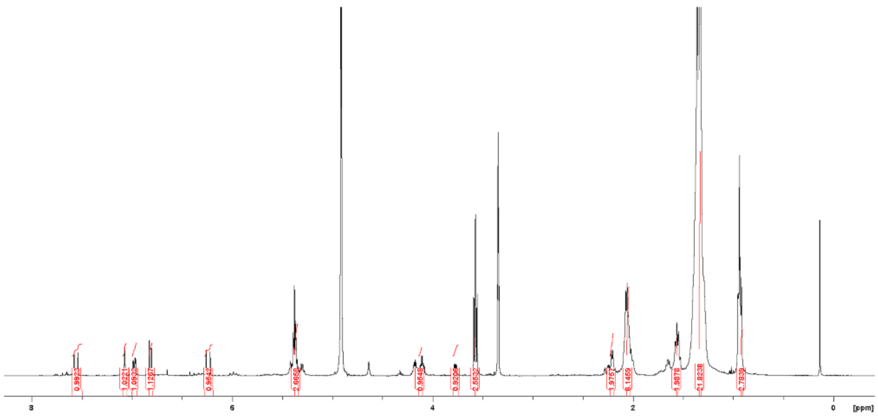

Figure S1. The ^{1}H NMR spectra of CGOA.

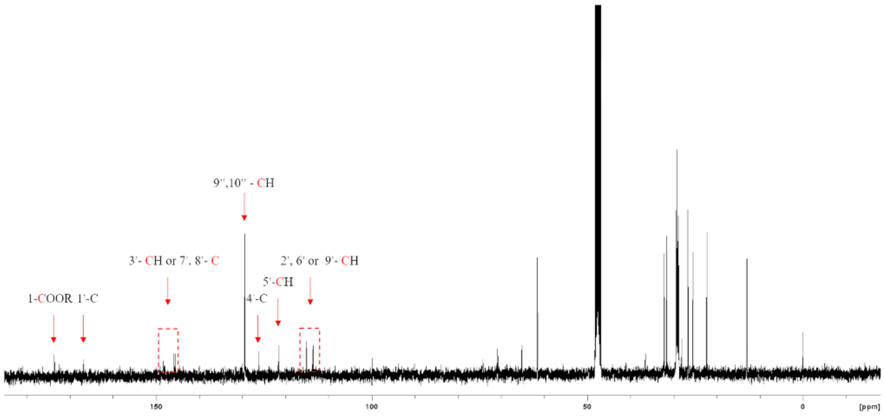

Figure S2. The ^{13}C NMR spectra of CGOA.

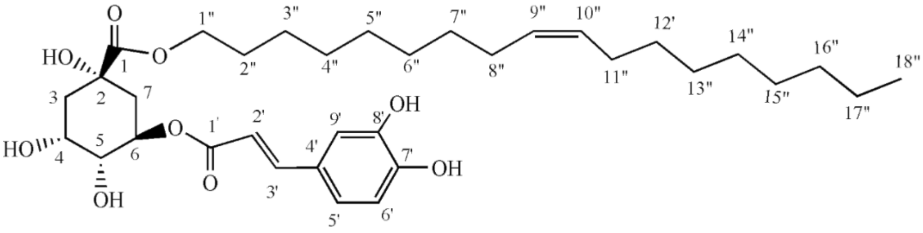

Figure S3. The chemical structure of CGOA.

Table S1. NMR characteristics of the ^{1}H and ^{13}C of CGA and CGOA.

No.	CGA		CGOA	
	^{1}H	^{13}C	^{1}H	^{13}C
1	—	175.62	—	173.63
2	—	72.04	—	99.99
3	2.23	37.35	2.21	36.62
4	4.19	69.87	4.12	65.29
5	3.75	74.72	3.76	74.24
6	5.35	70.57	5.36	70.82
7	2.07	36.81	2.04	32.28
1'	—	167.26	—	166.80
2'	6.28	113.84	6.22	113.74
3'	7.58	145.40	7.54	145.50
4'	—	126.39	—	126.20
5'	6.80	121.59	6.80	121.58
6'	6.97	115.07	6.96	115.12
7'	—	145.69	—	145.86
8'	—	148.18	—	148.34
9'	7.07	113.79	7.07	113.59
1"	—	—	3.56	61.61
2"	—	—	1.56	31.67
3"	—	—	1.33	25.54
4"	—	—	1.33	29.06
5"	—	—	1.33	29.37
6"	—	—	1.33	29.22
7"	—	—	1.33	28.92
8"	—	—	2.04	26.72
9"	—	—	5.36	129.44
10"	—	—	5.36	129.45
11"	—	—	2.04	26.74
12"	—	—	1.33	28.94
13"	—	—	1.33	29.42
14"	—	—	1.33	29.45
15"	—	—	1.33	29.25
16"	—	—	1.33	29.46
17"	—	—	1.33	22.35
18"	—	—	0.92	13.07

- Indicates that this C atom is not connected to -H, or not exist.

The difference in shifts of carbonyl atoms was underlined.
